# Multimodal cardioprotective strategy in cardiac surgery (the ProCCard trial): Study protocol for a multicenter randomized controlled trial

**DOI:** 10.1186/s13063-019-3638-3

**Published:** 2019-09-11

**Authors:** Pascal Chiari, Michel Durand, Olivier Desebbe, Marc-Olivier Fischer, Diane Lena-Quintard, Jean-Charles Palao, Catherine Mercier, Géraldine Samson, Yvonne Varillon, Matteo Pozzi, Nathan Mewton, Delphine Maucort-Boulch, Michel Ovize, Jean-Luc Fellahi

**Affiliations:** 1 Service d’Anesthésie-Réanimation, Hôpital Louis Pradel, Hospices Civils de Lyon, F-69677 Lyon, France; 2 Inserm U1060, Laboratoire CarMeN, IHU OPeRa, F-69394 Lyon, France; 3 Pole d’Anesthésie-Réanimation, Hôpital Albert Michallon, Centre Hospitalier Universitaire de Grenoble-Alpes, F-38043 Grenoble, France; 4 Service d’Anesthésie-Réanimation, Clinique de la Sauvegarde, Ramsay Générale de Santé, F-69009 Lyon, France; 5 Service d’Anesthésie-Réanimation, Centre Hospitalier Universitaire de Caen, F-14033 Caen, France; 6 Université de Normandie, UNICAEN, Caen, France; 7 Service d’Anesthésie-Réanimation, Institut Arnault Tzanck, F-06721 Saint Laurent du Var, France; 8 Service d’Anesthésie-Réanimation, Hôpital Nord, Centre Hospitalier Universitaire de Saint Etienne, F-42277 Saint Etienne, France; 9 Service de Biostatistique - Bioinformatique, Pôle Santé Publique, Hospices Civils de Lyon, F-69003 Lyon, France; 10 Université de Lyon, F-69000 Lyon, France; 11 Université Lyon 1, F-69100 Villeurbanne, France; 12 CNRS, UMR5558, Laboratoire de Biométrie et Biologie Évolutive, Équipe Biostatistique-Santé, F-69100 Villeurbanne, France; 13 Centre d’Investigation Clinique de Lyon (CIC 1407 Inserm), Hospices Civils de Lyon, F-69677 Lyon, France; 14 Service de Chirurgie Cardiaque, Hôpital Louis Pradel, Hospices Civils de Lyon, F-69677 Lyon, France; 15 Service d’Insuffisance Cardiaque, Lyon, France; 16 Service d’Explorations Fonctionnelles Cardiovasculaires, Lyon, France

**Keywords:** Cardioprotection, Cardiac surgery, Cardiopulmonary bypass, Preconditioning, Postconditioning, Multimodal strategy

## Abstract

**Background:**

Myocardial damage in patients undergoing cardiac surgery increases both morbidity and mortality. Different protective strategies dealing with either preconditioning or postconditioning or assessing a single aspect of cardioprotection have shown conflicting results. We tested the hypothesis that a multimodal approach would improve cardioprotection and limit myocardial damage following cardiac surgery with cardiopulmonary bypass.

**Methods:**

This study is a pragmatic multicenter (six French institutions), prospective, randomized, single-blinded, controlled trial. The randomization is stratified by centers. In the study, 210 patients scheduled for aortic valve surgery with or without coronary artery bypass grafting will be assigned to a control or a treatment group (105 patients in each group). In the control group, patients receive total intravenous anesthesia with propofol and liberal intraoperative blood glucose management (initiation of insulin infusion when blood glucose, measured every 60 min, is greater than 180 mg/dl), as a standard of care. The treatment group receives a bundle of care combining five techniques of cardioprotection: (1) remote ischemic preconditioning applied before aortic cross-clamping; (2) maintenance of anesthesia with sevoflurane; (3) tight intraoperative blood glucose management (initiation of insulin infusion when blood glucose, measured every 30 min, is greater than 140 mg/dl); (4) moderate respiratory acidosis (pH 7.30) at the end of cardiopulmonary bypass; and (5) a gentle reperfusion protocol following aortic unclamping. The primary outcome is myocardial damage measured by postoperative 72-h area under the curve of high-sensitivity cardiac troponin I.

**Discussion:**

The ProCCard study will be the first multicenter randomized controlled trial aiming to assess the role of a bundle of care combining several cardioprotective strategies to reduce myocardial damage in patients undergoing cardiac surgery with cardiopulmonary bypass.

**Trial registration:**

ClinicalTrials.gov, NCT03230136. Registered on July 26, 2017. Last updated on April 17, 2019.

**Electronic supplementary material:**

The online version of this article (10.1186/s13063-019-3638-3) contains supplementary material, which is available to authorized users.

## Background

Cardiac surgery with cardiopulmonary bypass (CPB) induces, consecutive to aortic clamping–unclamping, myocardial damage related to ischemia-reperfusion. The most common myocardial protection technique involves administration of a cardioplegic solute into the coronary arteries. Nevertheless, elevated postoperative cardiac troponin release remains a marker of poor prognosis after cardiac surgery [1, 2]. Many studies have, therefore, attempted to reduce myocardial ischemia-reperfusion injuries during CPB.

The protective effect of ischemic preconditioning was first described in 1986 by Murry et al. [3]. This process involves applying transient brief episodes of ischemia before subsequently sustained ischemia to render the myocardium more resistant to an ischemic insult. More recently, similar interventions applied at the onset of reperfusion, a process named postconditioning by analogy with preconditioning, have been described [4]. Since these first publications, a very large number of experimental studies have been carried out to characterize these phenomena and to understand their mechanisms. In a second stage, several clinical studies have attempted to reproduce these results in humans, with rather mixed results [5]. Most studies have been done in patients undergoing CPB. However, these specific clinical conditions, such as the major inflammatory processes induced by the heart–lung machine, the influence of the anesthetic agents, or the specificity of each cardiac surgical procedure, may complicate the interpretation of the results of such studies. Moreover, all the clinical studies that have been done to date have tested a single factor of myocardial protection, such as the administration of cyclosporine, the beneficial effects of anesthesia by means of halogenated volatile anesthetics, or the effect of remote ischemic preconditioning (RIPC) [6–8]. The biological effect of a single cardioprotective procedure might not be strong enough for translation into heterogeneous routine clinical situations, conversely to standardized experimental models.

The combination of multiple cardioprotective procedures offers the potential for increasing the biological and clinical effects of cardioprotection. Experimental work has found a synergistic effect of cardioprotection. However, no clinical study has sought to combine the beneficial effects of several techniques of cardioprotection in patients undergoing heart surgery with CPB [9, 10].

The present study evaluates the effect of a bundle of care combining several cardioprotective techniques during cardiac surgery. We seek to evaluate the effect and feasibility of adding different simple and patient-safe procedures compared to standard of care to reduce myocardial damage in patients undergoing CPB.

## Methods/design

### Design

ProCCard is a multicenter, prospective, randomized, controlled, single-blinded, two-arm study comparing a bundle of care combining five techniques of cardioprotection to a conventional approach in cardiac surgery with CPB. The trial is performed in accordance with the declaration of Helsinki (revised version, 2013), the European Guidelines for Good Clinical Practice (revised version, 2016), and the French laws. A checklist of recommended items to address in a clinical trial protocol according to the "Standard Protocol Items: Recommendations for Interventional Trials (SPIRIT 2013 Checklist)" is provided in Additional file 1. 

### Partners

Patients are recruited in six French institutions (details in Table 1). The study sponsor is the Direction of Clinical Research of the Hospices Civils de Lyon, a public academic institution in France. The Clinical Investigation Center of Lyon, an academic research organization within the Hospices Civils de Lyon (Inserm 1407), provides the methodological support, coordinates the trial, and also collects the trial data. All the analyses will be performed by the Biostatistics Department at the Hospices Civils de Lyon, which also provides methodological support to the study. The study is supported by the French Clinical Research Program 2016 (Programme Hospitalier de Recherche Clinique).

**Table 1:** ProCCard Investigators list

Site	Inclusion center	Investigators	Email address
01	Department of Anesthesiology and Intensive Care Medicine, University Hospital Louis Pradel, Hospices Civils de Lyon	Fellahi, Jean-Luc	jean-luc.fellahi@chu-lyon.fr
		Chiari, Pascal	pascal.chiari@chu-lyon.fr
		Joseph, Pierre	pierre.joseph@chu-lyon.fr
		Fornier, William	william.fornier@chu-lyon.fr
		Ferraris, Arnaud	arnaud.ferraris@chu-lyon.fr
02	Department of Anesthesiology and Intensive Care Medicine, Institut Arnault Tzanck, Saint Laurent du Var	Lena-Quintard, Diane	dianelena79@gmail.com
		Cady, Julien	juliencady@hotmail.com
		Causeret, Arnaud	acauseret@gmail.com
		Camarasa, Philippe	camarasa.p@wanadoo.fr
		Maccario, Michèle	m.maccario@tzanck.org
		de la Chapelle, Arnaud	a.delachapelle@tzanck.org
03	Department of Anesthesiology and Intensive Care Medicine, University Hospital, Grenoble	Durand, Michel	mdurand@chu-grenoble.fr
		Zunarelli, Romain	rzunarelli@chu-grenoble.fr
		Gaide-Chevronnay, Lucie	LGaide-Chevronnay@chu-grenoble.fr
		Marino, Maria Rosaria	MRMarino@chu-grenoble.fr
04	Department of Anesthesiology and Intensive Care Medicine, University Hospital, Saint Etienne	Palao, Jean-Charles	jean-charles.palao@chu-st-etienne.fr
		Grand, Nathalie	nathalie.grand@chu-st-etienne.fr
		Lanoiselee, Julien	julien.lanoiselee@chu-st-etienne.fr
05	Department of Anesthesiology and Intensive Care Medicine, Clinique de la Sauvegarde, Lyon	Desebbe, Olivier	oldesebbe@yahoo.com
		Delannoy, Bertrand	bertrand.delannoy@gmail.com
06	Department of Anesthesiology and Intensive Care Medicine, University Hospital, Caen	Fischer, Marc-Olivier	fischer-mo@chu-caen.fr
		Caspersen, Edouard	casperden-e@chu-caen.fr
		Cabart, Antoine	cabart-a@chu-caen.fr
		Ristovski, Robert	ristovski-r@chu-caen.fr
		Pottier, Véronique	pottier-v@chu-caen.fr
		Savary, Benoit	savary-b@chu-caen.fr

### Study population

#### Screening and inclusion

Patients are screened during the preoperative visit by the anesthesiologist, informed, and included after providing written consent.

#### Inclusion criteria

Patients scheduled for aortic valve surgery, with or without coronary artery bypass, older than 18 years, are eligible for enrollment.

#### Exclusion criteria

- Emergency surgery
- Redo surgery
- Preoperative treatment with nicorandil (an adenosine triphosphate-sensitive potassium channel opener), sulfonylurea, or repaglinide (two adenosine triphosphate-sensitive potassium channel blockers)
- Preoperative acute circulatory insufficiency justifying catecholamine support or mechanical circulatory assistance
- Severe chronic renal insufficiency (glomerular filtration rate < 30 ml/min)
- Severe chronic liver disease (spontaneous international normalized ratio > 2),
- Severe respiratory insufficiency (forced expiratory volume in 1 s < 40% theoretical value)
- Acute coronary syndrome less than 7 days old
- Current infection
- Peripheral arterial disease at upper limbs
- Any other surgical procedure associated with aortic valve surgery (combined valve surgery, Morrow’s myotomy, etc.)

### Perioperative procedure

Standard intraoperative monitoring consists of a five-lead electrocardiogram, frontal electroencephalography (BIS-monitor A2000®; Aspect Medical Systems, Norwood, MA, USA), pulse oximetry, peripheral arterial catheter, central pressure monitoring, capnography and vesical temperature probe.

Intravenous anesthesia is induced with either etomidate or propofol, sufentanil, or remifentanil (at the discretion of the investigator), and cisatracurium. The maintenance of anesthesia depends on the experimental group assignation. After systemic heparinization (300 IU/kg, activated clotting time > 400 s), the ascending aorta and right atrium are cannulated. A standard CPB with a disposable hollow-fiber membrane oxygenator is started with a target output of 2.4 l/min/m^2^ of body surface area. Surgery is performed under mild hypothermia (> 35 °C). After aortic cross-clamping, cardioplegia is achieved with crystalloid or blood solution, according to local protocols. After aortic unclamping, the heart is defibrillated if sinus rhythm does not resume spontaneously. Patients are transferred to the intensive care unit (ICU) and extubated when pressure support ventilation is tolerated.

### Experimental protocol

#### Randomization

Written informed consent is obtained for all patients before inclusion. Patients are randomly assigned to either the intervention or control group. Computer-generated randomized lists are drawn up by the Biostatistics Department of the Hospices Civils de Lyon before the start of the study. The randomization is stratified by i) centers and ii) individual need to perform coronary bypass during aortic valve surgery. The allocation is implemented in the electronic case report form.

#### Treatment group

In the treatment group, patients receive a bundle of care combining five techniques of cardioprotection (Table 2):

1. After intravenous induction, anesthesia is maintained during surgery with sevoflurane to induce both preconditioning and postconditioning. During CPB, sevoflurane is administered through the heart–lung machine only in two institutions (Lyon University Hospital and Arnault Tzanck Institute). In the four remaining centers, because the device for administering sevoflurane is not available on the heart–lung machine, propofol is administered during CPB.
2. RIPC is applied using a Riester® pneumatic tourniquet placed on the upper arm (Riester Company, 72,417 Jungingen, Germany). Three cycles of 5-min inflation to 200 mmHg/5 min deflation are performed after anesthesia induction and before skin incision.
3. Intraoperative blood glucose management involves the initiation of insulin infusion when blood glucose, measured every 30 min, is greater than 140 mg/dl.
4. During CPB, 5 min before aortic unclamping, blood gases are adjusted by limiting the flow of air/oxygen mixture in the heart–lung machine, in order to obtain an arterial blood pH lower than or equal to 7.30, and consequently to limit the “pH paradox” phenomenon occurring at the time of reperfusion [11, 12].
5. Just after aortic unclamping, a “gentle reperfusion” protocol is applied, consisting of a gradual recovery of the CPB flow (initial recovery of the flow at 20% of the theoretical flow, with an increase of 20% every 30 s, until a complete restoration of the CPB flow in 2 min) to limit reperfusion injuries.

**Table 2:** Perioperative protocol in each of the two treatment arms

	Control group	Treated group
Maintenance of anesthesia	Propofol	Sevoflurane
RIPC	No	Three cycles of: 5 min inflation to 200 mmHg/5 min deflation
Intraoperative blood glucose (BG) management	BG control every 60 min. Intravenous insulin infusion if BG greater than 180 mg/ml	BG control every 30 min. Intravenous insulin infusion if BG greater than 140 mg/ml
pH level at the end of CPB, before aortic unclamping	pH ≥ 7.40	pH ≤ 7.30
Gentle reperfusion	No	Gradual recovery of the CPB flow (initial recovery of the flow at 20% of the theoretical flow, with an increase of 20% every 30 s, until complete restoration in 2 min)

#### Control group

In the control group, patients are managed according to a standard of care (Table 2):

1. Anesthesia is maintained with continuous infusion of propofol during surgery (halogenated volatile anesthetics are not allowed in this group).
2. Intraoperative blood glucose management involves the initiation of insulin infusion when blood glucose, measured every 60 min, is greater than 180 mg/dl.
3. During CPB, 5 min before aortic unclamping, blood gases are adjusted by modifying the flow of air/oxygen mixture on the heart–lung machine to obtain an arterial blood pH higher than or equal to 7.40.
4. Just after aortic unclamping, the theoretical CPB flow is restored at the earliest, according to the clinical tolerance of the patient (no gentle reperfusion).

### Outcomes

The primary outcome is the postoperative 72-h area under the curve (AUC) for high-sensitivity cardiac troponin I (hsTnI) release. Blood samples are collected after induction of anesthesia (baseline) and 4, 8, 12, 24, 48, and 72 h after aortic unclamping (Table 3). All samples are frozen carefully and sent to a centralized laboratory for measurement (see below).

**Table 3:** ProCCard trial schedule during the study period

	Preoperative day	Day of surgery				Postoperative day	Hospital discharge	Postoperative day
		Baseline	Before aortic unclamping	ICU arrival	H4-H8-H12	H24-H48-H72		Day 30
Eligibility screen	x							
Informed consent	x							
Randomization		x						
High-sensitivity troponin I		x			x	x		
Blood glucose level		x	x	x				
Arterial lactate		x		x	x (H8)			
Arterial pH			x	x				
Creatininemia	x			x		x		
ECG	x			x		x		
Trans-thoracic echocardiography	x						x	
Quality of life	x							x
Visit before hospital discharge							x	
Phone call								x
Adverse events		x	x	x	x	x	x	x

The secondary outcomes are hsTnI level (peak and 24 h after aortic unclamping), extubation time, length of stay in ICU and hospital, Index Gravity Score (IGS II; the scoring system measuring the severity of disease for patients admitted to ICU), the three-level version of EQ-5D (EQ-5D-3 L) health status score (comparison between preoperative and 30 day postoperative), and the need for catecholaminergic support [13]. Major adverse events occurring during 30 postoperative days are collected and reported as follows: all-cause of death, serious infection requiring antibiotic therapy, major bleeding requiring transfusion of ≥ 5 U packed red blood cells or surgical intervention, respiratory insufficiency, stroke, arrhythmia requiring medical therapy.

### Data collection

#### Baseline data

The following data are collected in the preoperative period: initial valvular aortic pathology requiring surgical correction, concomitant pathology (hypertension, dyslipidemia, diabetes, stroke, atrial fibrillation, chronic obstructive pulmonary disease, smoking), and medications (β-blockers, nitrates, statins, Ca^2+^ channel blockers, angiotensin-converting enzyme inhibitor, angiotensin II receptor blocker, diuretics, platelet inhibitors, insulin, oral antidiabetic drugs, amiodarone, heparin, oral anticoagulant therapy), age, sex, body mass index, NYHA class, ASA class, Logistic EuroSCORE I, blood creatinine level, blood hemoglobin level, electrocardiogram, EQ-5D-3 L health status score, echocardiography (left ventricular (LV) ejection fraction, LV mass index calculated using LV end-diastolic diameter, LV end-diastolic posterior wall thickness, LV end-diastolic interventricular wall thickness).

#### Intraoperative data

During surgery, the following data are collected: CPB duration (min), aortic cross-clamping duration (min), type and volume of cardioplegia (ml), surgical procedure performed, baseline hsTnI level, baseline lactate level, total IV sufentanil/remifentanil (μg), total IV propofol (mg), total IV insulin (IU), catecholaminergic treatment, blood glucose concentrations during the operative period, arterial blood gas harvested 5 min before aortic unclamping.

#### Postoperative data

The following data are also collected (Table 3): blood lactate (ICU arrival, and 8 h after aortic unclamping) and creatinine (ICU arrival, 24, 48, and 72 h after aortic unclamping) levels, electrocardiogram (ICU arrival, 24, 48, and 72 h after aortic unclamping), extubation time, SAPS II, length of stay in ICU and hospital, echocardiography at 5-day postoperative (LV ejection fraction, LV mass index), EQ-5D-3 L health status score at 30 day postoperative (phone call), any adverse event occurring during this period.

#### Centralized analysis of hsTnI

Blood samples for the analysis of hsTnI are collected in each institution, centrifuged at 2000 g for 10 min. The supernatant is removed and stored in a freezer (− 80 °C) before transfer for centralized analysis to the Biochemical Laboratory at the Lyon University Hospital (ARCHITECT STAT Troponin I; Abbott, North Chicago, IL, USA).

#### Sample size

The sample size calculation was performed according to hypotheses stemming from a previous study carried out in the department of cardiac anesthesia of the same facility [6]. One hundred patients per arm would ensure an 80% power of detecting a decrease in the AUC of hsTnI release within the three first postoperative days (AUC of 242 in the control arm and 169 in the treatment arm) considering a common standard deviation of 183 and a two-sided Student test. Assuming a 5% rate of missing data, there should be 105 patients in each arm; thus, 210 patients are to be included in the study.

#### Statistical analyses

The main analysis will be carried out within the intention-to-treat (ITT) population. The ITT population is defined as all patients included in the study whatever the selection criteria, the treatment strategy, or the adequacy for evaluation. All missing data necessary for the calculation of the outcome criterion (AUC of serum level of hsTnI within 3 days after surgery) will be imputed. The causes and contexts of missingness will orient the choice of the imputation method. The main analysis will compare the AUC of serum level of hsTnI within 3 days after surgery between the treatment and the control arm using a mixed-effect generalized linear model. According to the recommendations of the international conference on harmonization of technical requirements for the registration of pharmaceuticals for human use (ICH), each variable used for the stratification of the randomization will be introduced in the model as a random effect or fixed effect. Variable “arm” will be introduced as a fixed effect. Statistical significance on two-sided Wald test will be set at 0.05.

The same analysis will be carried out on the per-protocol (PP) population. The PP population is defined as all patients of the ITT population with no major deviation from the study protocol. This analysis will consider the actually applied strategy. A sensitivity analysis will be carried out after excluding all patients who experienced, within 3 days after surgery, any event able to bias the analysis of the main outcome. The main outcome will also be analyzed using a mixed-effect generalized linear model to investigate the effect of patient management through considering various practices likely to influence hsTnl kinetics, especially glycemic and lactic acidosis monitoring.

All secondary outcomes will be summarized, per arm, according to their nature (qualitative or quantitative). The peak of serum level of hsTnI and the level of hsTnI at 24 h after aortic unclamping will be analyzed in the same way as the main outcome. The duration of mechanical ventilation and the duration of stay in the ICU will be similarly analyzed either after log-transformation of the values or using a survival analysis technique (time-to-event analysis of the time to mechanical ventilation discontinuation or the time to discharge from hospital, respectively). A frailty Cox model may be used to allow for the variables used for stratification. The length of hospital stay will be analyzed with a Cox frailty model adjusted for the variables used for stratification. The IGS II will be analyzed in the same way as the main outcome after checking the score distribution. If necessary, an adequate score transformation may be applied.

The need for catecholaminergic support will be analyzed with a logistic model adjusted for the variables used for stratification.

The occurrence of at least one postoperative complication will be analyzed with a logistic model adjusted for the variables used for stratification. The complications considered are: post-operative hemorrhage, need for postoperative blood transfusion, the need for revision surgery for bleeding, the occurrence of respiratory insufficiency requiring intubation or not, postoperative infection, neurological disorder, and complete arrhythmia by atrial fibrillation. The postoperative adverse events will be analyzed in the safety population. The safety population is defined as all patients of the ITT population who received any treatment of the actually applied strategy. The proportions of patients concerned by the secondary outcomes will be calculated by arm and type of complication.

The EQ-5D-3 L score will be analyzed using a mixed linear regression model that will include a variable for “time” (at 30 days after surgery vs before surgery), a variable for “group”, and an interaction between these two variables. This model will also be adjusted for the variables used for stratification. The coefficient of interaction will be tested to compare the arms. The score distribution will be examined for floor and/or ceiling effects. If necessary, a Tobit model will be used. Survival will be analyzed with a Cox model adjusted for the variables used for stratification.

### Regulatory issues

The study has been approved by the French National Agency for Medicines and Health Products Safety (Agence Nationale de Sécurité du Médicament et des Produits de Santé, 93,285 Saint-Denis, France) on August 16, 2017 (ID-RCB: 2017-A00694–49) and by an independent ethics committee (Comité de Protection des Personnes, Ile-de-France VII, 94270 Le Kremlin Bicêtre, France) on September 13, 2017. The study was registered on ClinicalTrials.gov on July 26, 2017 (NCT03230136). Central ethical approval has been confirmed from Comité de Protection des Personnes (reference approval N° 2017-A00694–49) and we will not begin recruiting at other centers in the trial until local ethics approval has been obtained. Patient data are collected anonymously on the electronic platform. All severe adverse events are documented in the electronic case report form and declared to the Pharmacovigilance Risk Assessment Committee (Hospices Civils de Lyon). An independent Data Safety Monitoring Board including three external experts (Pr. Claude Girard, anesthesiologist, Dijon, France, Pr. Julien Amour, anesthesiologist, Paris, France, and Dr. Remy Morello, biostatistician, Caen, France) will conduct safety monitoring.

## Discussion

Cardioprotective strategies of preconditioning and postconditioning during cardiac surgery with CPB have shown very promising and consistent results in experimental models and small clinical studies. In larger groups of patients in randomized studies, however, these cardioprotective strategies have failed to show any significant clinical benefit [3–5]. Experimental studies clearly demonstrated the benefit of halogenated volatile anesthetics before ischemia (halogen-induced preconditioning) and at the onset of reperfusion (halogen-induced postconditioning) [9, 14]. De Hert et al. [7] showed in patients undergoing coronary artery surgery the cardioprotective effects of sevoflurane when administered throughout the operation. Unfortunately, we and others did not find such a protective effect of halogenated volatile anesthetics in other clinical studies [15, 16]. RIPC is another example of this recurring problem. Initially, Hausenloy et al. [8] demonstrated that RIPC reduced serum troponin T release in patients undergoing coronary artery bypass graft surgery. Similarly, Zarbock et al. [17] reduced the rate of perioperative acute kidney injury by applying RIPC in patients undergoing cardiac surgery. Unfortunately, ERICCA and RIPHeart, two large-scale multicenter trials, did not confirm the previous results [18, 19].

These results of disappointing clinical studies raise questions because most of the experimental works in standardized models are positive. In addition, the human myocardium, under experimental conditions, is just as sensitive to preconditioning as other species [20]. Nevertheless, it must be kept in mind that clinical conditions, especially during cardiac surgery, are very different from experimental studies. In a clinical setting, many confounders can affect the efficacy of cardioprotective strategies. Many surgical procedures are performed in older patients and the protective effects of conditioning decline with age [21]. The preoperative drug treatments prescribed to patients can also interfere with the cellular mechanisms of conditioning. The major inflammatory response to CPB is another source of confusion [22]. Finally, postoperative serum troponin release, which is often the primary outcome of the clinical studies, is also related to the type of cardiac procedure [23].

On the other hand, clinical studies have focused on only one conditioning factor, such as the effect of cyclosporine administration, the potential beneficial effect of halogenated volatile anesthetics, or the protective effect of RIPC. However, experimental studies have highlighted that a synergy exists between the different conditioning triggers. We previously demonstrated that the combination of a concentration of halogenated volatile anesthetics that did not produce cardioprotection alone was able to reduce the time threshold required for ischemic postconditioning [9]. There is also a link between hyperglycemia, a phenomenon commonly encountered during cardiac surgery, and the preconditioning effect of halogenated volatile anesthetics [24, 25]. Beneficial effects of simvastatin, when added to ischemic preconditioning, restored cardioprotection even in hyperglycemic animals [10]. So, it seemed important to carry out a study evaluating the interest of combining several cardioprotective factors in order to develop an intraoperative cardioprotective strategy.

The biological effect of a single cardioprotective procedure might not be strong enough for translation into heterogeneous routine clinical situations compared to standardized experimental models. The combination of multiple cardioprotective procedures offers the potential for increasing the biological and clinical effects of cardioprotection.

All the cardioprotective procedures we are combining in this protocol were chosen carefully. The purpose was to combine easily applicable procedures with potential synergistic effects. As mentioned by previous studies, the beneficial effect of RIPC is potentially amplified during halogenated anesthesia. Kottenberg et al. [26] conducted a study during coronary artery bypass graft surgery. Three cycles of 5 min upper arm ischemia/5 min reperfusion decreased the troponin AUC by 50% during isoflurane anesthesia but had no effect under propofol anesthesia. The same results were confirmed later in a larger study [27]. We therefore decided that patients in the “treated group” would be anesthetized with sevoflurane and would also benefit from a RIPC protocol. In parallel, the frequency of perioperative dysglycemia is underestimated in cardiac surgery and its impact on postoperative morbidity and mortality is unquestionable [24]. Because hyperglycemia can impair the myocardial tolerance to ischemia-reperfusion and knowing that insulin is cardioprotective, we included these parameters in the bundle of care of the ProCCard study, in conjunction with RIPC and sevoflurane [28]. Based on the study of Duncan et al. [29], we chose to keep the intraoperative blood glucose threshold at 8 mmol/l in order to avoid any hypoglycemia. Our objective was to control the patient’s glycemia, rather than to conduct a hyperinsulinemic management protocol, as recently proposed [30]. The moment of reperfusion, which corresponds to the aortic unclamping, is also a double-edged sword. Temporary acidosis at the time of reperfusion can limit infarct size by delaying the opening of the mitochondrial permeability transition pore [11]. We have therefore ensured that the pH level is in the low range of clinically acceptable values to limit the “pH paradox” phenomenon [12, 31]. The final procedure in this bundle of care is a gentle reperfusion protocol. Controlled reperfusion after an ischemic period protects the myocardium, probably by the activation of the phosphatidylinositol 3-kinase–Akt pathway [32]. We therefore decided to limit reperfusion injuries by a gradual restoration of the theoretical CPB flow over 2 min.

In conclusion, the ProCCard study will be the first multicenter randomized controlled trial aiming to assess the role of a bundle of care combining several cardioprotective procedures to reduce myocardial damage in patients undergoing CPB.

## Trial status

The ProCCard trial is currently recruiting patients (protocol version 3, September 3, 2018). The first patient was included on January 3, 2018. The expected inclusion period is 24 months. Recruitment will be completed approximately at the end of 2019.

## Additional file

Additional file 1: SPIRIT 2013 Checklist: Recommended items to address in a clinical trial protocol and related documents. (DOC 121 kb)

## Data Availability

The datasets analyzed during the current study are available from the corresponding author on reasonable request.

## Abbreviations

AUC: Area under the curve
CPB: Cardiopulmonary bypass
hsTnI: High-sensitivity cardiac troponin I
ICU: Intensive care unit
IGS II: Index Gravity Score
ITT: Intention-to-treat
LV: Left ventricular
PP: Per-protocol
RIPC: Remote ischemic preconditioning
