# Supplementary material for: Multimodal cardioprotective strategy in cardiac surgery (the ProCCard trial): Study protocol for a multicenter randomized controlled trial
Source: Trials. 2019 Sep 11;20:560. doi: 10.1186/s13063-019-3638-3 (PMC6737694; doi:10.1186/s13063-019-3638-3)
Supplement: Supplementary file 1 — SPIRIT 2013 Checklist: Recommended items to address in a clinical trial protocol and related documents. (DOC 121 kb) [file 13063_2019_3638_MOESM1_ESM.doc]

**Allocation**

**Post-operative Follow-Up from day 1 to day 30**

**Enrollment**

**Informed consent**

Patients scheduled for aortic valve surgery,

with or without coronary artery bypass.

Exclusion :

♦  Emergency Surgery.

♦  Redo Surgery.

♦  Preoperative treatment with nicorandil, sulfonylurea or repaglinide.

♦  Preoperative shock state.

♦  Severe chronic renal insufficiency, liver disease or respiratory insufficiency.

♦  Acute coronary syndrome less than seven days old.

♦  Current infections.

♦  Peripheral arterial disease at upper limbs.

♦  Any other surgical procedure associated to aortic valve surgery

(combined valve surgery, Morrow’s myotomy…).

Allocated to Control strategy (n=105):

♦ Maintenance of anesthesia = Propofol.

♦ Remote Ischemic Preconditioning = No.

♦ IV insulin infusion if blood glucose, controlled every 60 min, is greater than 180 mg/ml.

♦ pH>7.40 at reperfusion.

♦ No gentle reperfusion.

Allocated to Protective strategy (n=105):

♦ Maintenance of anesthesia = Sevoflurane.

♦ Remote Ischemic Preconditioning = 3 cycles.

♦ IV insulin infusion if blood glucose, controlled every 30 min, is greater than 140 mg/ml.

♦ Moderate acidosis (pH<7.30) at reperfusion.

♦ Gentle reperfusion.

Inclusion of 210 patients.

The randomization is stratified by:

♦  Centers.

♦  Individual need to perform coronary artery bypass during aortic valve surgery.
